# Malnutrition, Inflammation, Atherosclerosis Syndrome (MIA) and Diet Recommendations among End-Stage Renal Disease Patients Treated with Maintenance Hemodialysis

**DOI:** 10.3390/nu10010069

**Published:** 2018-01-11

**Authors:** Małgorzata Maraj, Beata Kuśnierz-Cabala, Paulina Dumnicka, Agnieszka Gala-Błądzińska, Katarzyna Gawlik, Dorota Pawlica-Gosiewska, Anna Ząbek-Adamska, Małgorzata Mazur-Laskowska, Piotr Ceranowicz, Marek Kuźniewski

**Affiliations:** 1 Faculty of Medicine, Dietetics, Jagiellonian University Medical College, Anny St. 12, 31-008 Kraków, Poland; malgorzatamaraj@gmail.com; 2 Department of Diagnostics, Jagiellonian University Medical College, Kopernika 15A St., 31-501 Kraków, Poland; mbkusnie@cyf-kr.edu.pl (B.K.-C.); k.gawlik@uj.edu.pl (K.G.); dorota.pawlica@uj.edu.pl (D.P.-G.); 3 Department of Medical Diagnostics, Faculty of Pharmacy, Jagiellonian University Medical College, Medyczna 9 St., 30-688 Kraków, Poland; paulina.dumnicka@uj.edu.pl; 4 Dialysis Therapy Centre, St’ Queen Jadwiga Clinical District Hospital No. 2, Lwowska St. 60, 35-301 Rzeszów, Poland; agala.edu@gmail.com; 5 Faculty of Medicine, University of Rzeszów, Kopisto Ave. 2a, 35-310 Rzeszów, Poland; 6 Diagnostic Department, University Hospital in Krakow, Kopernika 15B St., 31-501 Kraków, Poland; azabek@su.krakow.pl (A.Z.-A.); mbmazur@cyf-kr.edu.pl (M.M.-L.); 7 Department of Physiology, Jagiellonian University Medical College, Grzegórzecka 16 St., 31-531 Kraków, Poland; 8 Department of Nephrology, Jagiellonian University Medical College, Kopernika 15C, 31-501 Kraków, Poland; marek.kuzniewski@uj.edu.pl

**Keywords:** diet, chronic kidney disease, inflammation, hemodialysis

## Abstract

Malnutrition-inflammation-atherosclerosis syndrome is one of the causes of increased mortality in chronic kidney disease (CKD). The aim of the study was to assess the inflammation and nutritional status of patients in end-stage kidney disease treated with maintenance hemodialysis. The study included a group of 98 hemodialyzed patients with stage 5 CKD (38 women and 60 men). Albumin, prealbumin (PRE), and C-reactive protein (CRP) were measured in serum samples collected before mid-week dialysis. Fruit and vegetables frequency intakes were assessed with a questionnaire. CRP was above the reference limit of 5 mg/L in 53% of patients. Moreover, the Glasgow Prognostic Score (GPS) indicated the co-occurrence of inflammation and protein calorie malnutrition in 11% of patients, and the presence of either inflammation or malnutrition in 25%. The questionnaire revealed that hemodialyzed patients frequently exclude fruit and vegetables from their diets. Nearly 43% of the interviewed patients declared frequently eating vegetables, and 35% declared frequently eating fruit, a few times per week or less. The most frequently selected fruit and vegetables had a low antioxidant capacity. The strict dietary restrictions in CKD are difficult to fulfill, and if strictly followed, may lead to protein-calorie malnutrition.

## 1. Introduction

One of the most serious clinical issues encountered in end-stage renal disease (ESRD) patients is the co-occurrence of malnutrition, inflammation, and atherosclerosis (MIA syndrome). The prevention of MIA syndrome is of utmost importance, because the emergence of each of its components worsens the survival potential of ESRD patients [1]. In response to high mortality and low quality of life, improving patients’ health by introducing an adequate diet and encouraging physical activity becomes one of the most important therapeutic goals [2,3,4]. 

Normal kidneys maintain a stable acid-base balance. Chronic kidney disease (CKD) progression leads to reduced excretion of bicarbonate and hydrogen ions [5]. Macrophages exposed to acidic uremic environment stimulate the production of tumor necrosis factor-α (TNF-α), initiating the acute phase response and leading to an increased concentration of C-reactive protein (CRP), which correlates with metabolic acidosis [6]. However, the decrease of TNF-α can be observed alongside the restoration of the acid-base balance in hemodialysis patients [7].

In stage 5 CKD, dietary restrictions such as the elimination of products rich in potassium, sodium, phosphates, and sulfur-containing amino acids are indicated [8]. However, patients often find it difficult to choose a permissible food product, and either do not comply with the restraints and categorically reject dietary guidelines, or they zealously adhere to the diet while eliminating most food products, and in consequence do not meet the required energy intake.

Koueiry et al. [9] have shown that 97% of hemodialysis patients consumed less than 25 g of dietary fiber per day. Another study has revealed that dialysis patients present a lower intake of vitamin C and the carotenoids lycopene, cryptoxanthin, and lutein than controls [10]. Dietary recommendations for stage 5 CKD patients put the strongest emphasis on restricting the potassium that is found in abundance in fruit and vegetables. However, in the face of elevated oxidative stress and increased cardiovascular risk, eliminating sources of antioxidants i.e., fruit and vegetables, seems to be unwarranted [9]. The adequate caloric (35 kcal/kg/day) and protein intake (1.2 g/kg) is recommended to prevent malnutrition [11]. The recommendations pose a difficult dilemma, because almost any increase in the caloric intake, especially increased protein and carbohydrate supply, results in the concomitant increase in potassium or phosphorus in the diet [12]. A constantly elevated concentration of serum phosphorus can lead to secondary hyperparathyroidism, renal osteodystrophy, and cardiovascular calcification; therefore, the reduction of the phosphorus intake to 10–12 mg/g protein for hemodialysis patients is recommended [13]. However, it is important to remember that the choice of low-phosphorus products is to a large extent in contradiction with the protein demand. The authors of key reviews concerning dietary guidelines for hemodialysis patients pose a question as to what extent the above numeric values are still valid, and they suggest turning towards more patient-oriented dietary recommendations [8,9,14]. Kalantar-Zadeh et al. [14] propose to lessen most restrictions and accept a more balanced and individualized approach, as the beneficial effect of most dietary restrictions has not been explicitly confirmed. Meanwhile, their strict compliance can significantly decrease a patient’s quality of life. When prescribing a diet, it is important to consider that ESRD patients develop an adaptive mechanism to increased potassium intake, and that pharmacologic treatment is able to diminish the harmful effects of calcium-phosphate disturbance. 

The aim of the study was the evaluation of the association between the lifestyle habits, including fruit and vegetables intake, on inflammation and the nutritional status of ESRD patients treated with maintenance hemodialysis, as well as the subsequent reconsideration of dietary guidelines in this group of patients.

## 2. Material and Methods

### 2.1. Patients

The study included adult patients with stage 5 CKD treated with maintenance hemodialysis (3–5-h dialysis sessions three times a week). The patients were recruited at two centers: the Department of Nephrology of Jagiellonian University Medical College in Kraków, and the Dialysis Therapy Centre of Clinical District Hospital No. 2 in Rzeszów in December 2016 and January 2017. The inclusion criteria were: informed consent for participation, and a stable course of the disease for at least three months. Patients with signs and symptoms of acute inflammation, and those with intellectual or mental disabilities that made them unable to understand and answer the questionnaire, were excluded. Moreover, we did not enroll patients with ESRD in the course of systemic connective tissue disease and patients with disseminated neoplastic disease. As the questionnaire was provided in the Polish language, the study required that the participants understand and speak Polish. Patients with a history of renal transplantation were included in the study provided that the disease course was stable for the last three months, no signs and symptoms of transplant rejection were present, and there was no more need for immunosuppressive treatment.

During the study period, all of the patients treated in both dialysis centers were middle European Caucasians.

### 2.2. Study Design

The patients’ dietary and lifestyle habits, as assessed with the questionnaire described below, were treated as the exposure variables. The answers to the questionnaire were compared with the laboratory data, which was obtained once during the same week. The laboratory markers of malnutrition and inflammation were treated as the main outcome variables. Demographic data were based on questionnaire and verified with patients’ records. The data regarding disease course, treatment, and comorbidities were assessed based on patients’ records at the dialysis centers. Demographic data such as sex and age were the main covariates in the analysis. 

### 2.3. Questionnaire

The dietary intake of fruit and vegetables was assessed with a self-constructed questionnaire consisting of 22 questions. The questionnaire inquired about gender, age, body mass, height, place of residence, period of dialysis, comorbidities, and lifestyle i.e., level of physical activity, amount of sleep, and dietary supplement use. Patients were asked about overall average fruit and vegetables consumption frequency in the preceding year. They selected the most appropriate answer among the following: >2 portions per day, 1–2 portions per day, a few portions per week, <1 portion per week. We have asked about fruit and vegetables processing, including cooking, cooking with double-water change, baking, etc. Moreover, patients were asked about the intake of particular 13 fruits and 14 vegetables most frequently used in middle-eastern Europe, including those with high antioxidant capacity. The possible answers to the detailed questions were the following: never, a few times per year, 1–3 times per month, a few times per week, once a week, once a day. Based on the answers, we calculated the number of portions of the particular fruit and vegetables per year. 

The patients were interviewed during their routine hemodialysis sessions at an outpatient dialysis unit in Kraków and Rzeszów. The questionnaire has been completed by the patients on their own or with author’s assistance (M.M.) whenever needed.

### 2.4. Laboratory Tests

The complete blood counts and serum biochemistry tests were performed as a part of the routine monitoring of patients, using samples collected before the mid-week dialysis session. Complete blood counts, serum urea, sodium, potassium, calcium, and phosphate were available for all patients, while iron, latent iron binding capacity (UIBC), and total iron binding capacity (TIBC) were only available for patients treated in Kraków, and lipid profiles were only available for those treated in Rzeszów. This was due to local differences in routine patients’ monitoring. 

The additional aliquots of sera were collected to measure parameters associated with malnutrition and inflammation, i.e., prealbumin (PRE), albumin, and C-reactive protein (CRP). CRP/PRE ratio and the Glasgow Prognostic Score (GPS) based on serum albumin and CRP results, were also calculated [15].

### 2.5. Ethics

Participation in the study was voluntary, and each patient gave his/her written consent to take part in the study. The study had the consent of the Bioethics Committee of the Regional Medical Chamber in Rzeszów, Poland (approval number 70/2014/B issued on 19 September 2014). 

### 2.6. Statistical Analysis

The number of patients/percentage of the study group were reported for categories. Mean ± standard deviation and median (lower-upper quartile) were shown for the quantitative variables with normal and non-normal distribution, respectively. The differences between groups were assessed with the *t*-Test or Mann-Whitney test, according to distribution. Contingency tables were analyzed with the chi-squared test. The Spearman rank coefficient was used to study correlations. Simple logistic regression was used to study associations between the results of the questionnaire and laboratory tests, reflecting malnutrition or inflammation. The predictors that were statistically significant in simple analysis were further investigated in multiple logistic regression. Results were considered significant at *p* < 0.05. The analysis was performed with Statistica 12.0 (StatSoft, Tulsa, OK, USA) software. 

## 3. Results

The study comprised 98 ESRD patients (38 women and 60 men) aged 19–96 years. Overall, 54 patients were recruited at the Department of Nephrology of Jagiellonian University Medical College in Kraków, and 44 patients at the Dialysis Therapy Centre of Clinical District Hospital No. 2 in Rzeszów in December 2016 and January 2017. The participants were treated with maintenance hemodialysis due to diabetic kidney disease (69%), chronic glomerulonephritis (16%), hypertensive nephropathy (6%), polycystic renal disease (3.5%), congestive nephropathy, and congenital defects of the urinary tract (2.5%). Thirteen percent of patients had a history of previous renal transplantation. Among the 98 patients, 72% were treated for renal anemia with erythropoiesis stimulating agents (ESA): darbepoetin alfa (42%), methoxy polyethylene glycol-epoetin beta (34%), epoetin alfa (19%), and epoetin beta (5%).

The demographic characteristics of the study group are presented in Table 1. Mean body mass index (BMI) values in the studied group were about the upper normal values (Table 1). The answers to the lifestyle questions of the questionnaire revealed that nearly 1/5 of patients were active smokers, none admitted high physical activity, and about 1/3 of patients used dietary supplements (Table 1). There were no significant differences between men and women regarding clinical and demographic characteristics (except for body mass), as well as lifestyle characteristics (smoking, duration of sleep, physical activity) (Table 1). The analysis of the fruit and vegetables intake disclosed that 43% of the patients reported eating vegetables, and 36% reported eating fruit only a few times per week or less frequently. No sex-related differences were observed in overall vegetable consumption; however, women reported more frequent overall fruit consumption than men (Table 1). However, there were no significant differences between men and women regarding the choice of fruit or vegetables. The participants consumed mainly cooked vegetables, although only the minority adopted water change during cooking (Table 1). No correlations were observed between fruit and vegetables consumption or processing, and the serum concentrations of potassium or phosphate.

No patients presented with a high degree of malnutrition, according to the concentrations of albumin and prealbumin (Table 2). However, 53% of patients had CRP above the reference limit of 5 mg/L. Moreover, the GPS revealed the co-occurrence of inflammation and protein-caloric malnutrition in 11% of patients, while either inflammation or malnutrition were detected in 25%. On average, women had lower serum albumin and prealbumin, and higher CRP concentrations. However, only albumin concentrations differed significantly between sexes (Table 2). The studied parameters of malnutrition and inflammation were highly correlated with each other (Table 3). However, no correlations were observed between serum albumin, prealbumin, CRP, CRP/PRE, or GPS and total cholesterol, and only albumin was significantly positively correlated with hemoglobin (Table 3).

**Figure 1:**
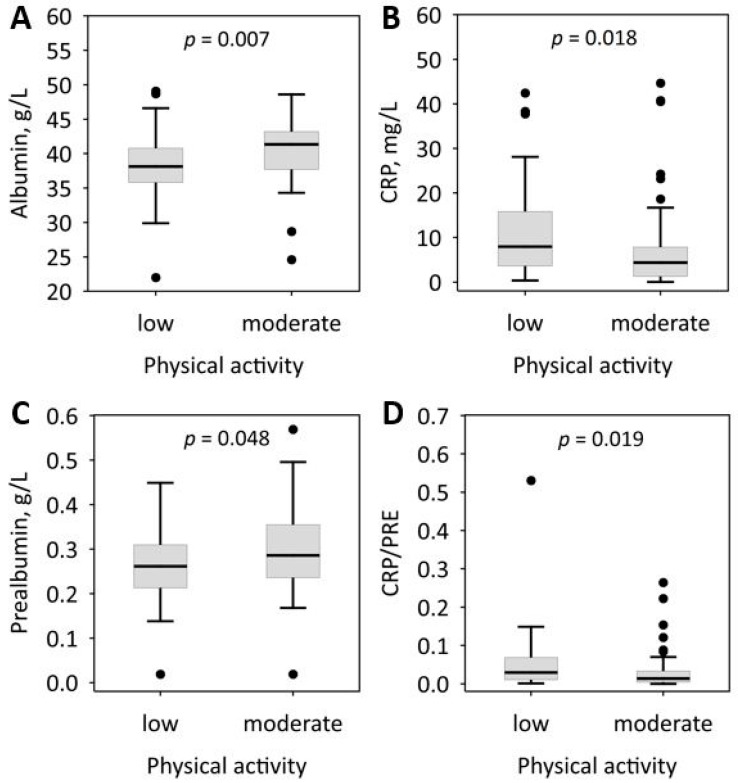
Associations between the level of physical activity reported by patients and the selected markers of malnutrition and inflammation: albumin (**A**); C-reactive protein (CRP) (**B**); prealbumin (**C**); and C-reactive protein to prealbumin ratio (CRP/PRE) (**D**). Data are shown as median, lower-upper quartile (box), non-outlier range (whiskers), and outliers (dots).

We did not observe statistically significant correlations between the frequency of consumption of fruit or vegetables and the serum concentrations of albumin, prealbumin, CRP, CRP/PRE, or the results of GPS. In simple logistic regression, the consumption of one or more portions of fruit per day was significantly negatively associated with low serum albumin, while no other significant relationships were found between fruit and vegetables consumption and malnutrition or inflammation (Table 4). Among the studied lifestyle characteristics, moderate physical activity was associated with higher serum albumin and prealbumin, as well as lower CRP and CRP/PRE (Figure 1). Also, physical activity was a significant predictor of CRP/PRE above the median value of 0.019, as well as GPS values of 1 or 2 in simple logistic regression (Table 4). However, these associations were not confirmed after adjustment for age (Table 4).

**Figure 2:**
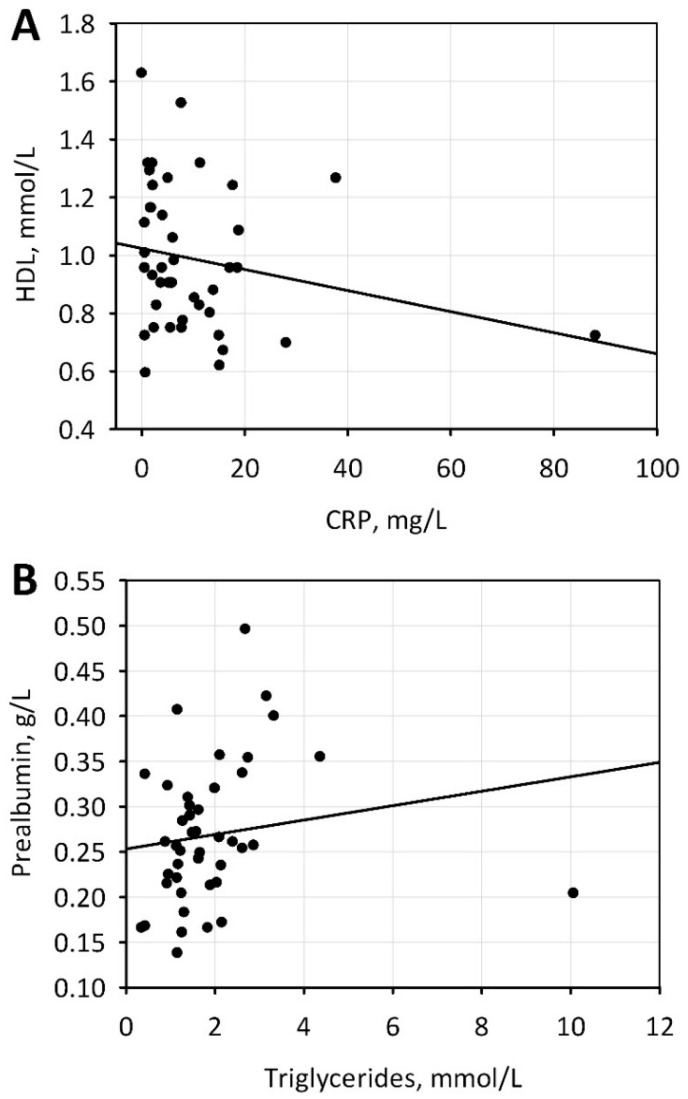
Correlations between lipid markers and selected markers of malnutrition and inflammation: HDL-cholesterol and CRP (**A**), triglycerides and prealbumin (**B**). HDL: high-density lipoprotein; CRP: C-reactive protein.

Laboratory tests confirmed anemia in most patients (Table 5). Electrolyte disturbances were also common, with hyperkalemia in nearly 50% and hyperphosphatemia in more than 80% of patients (Table 5). Moreover, the substantial proportion of hemodialyzed patients had dyslipidemia (Table 5). Higher concentrations of triglycerides were associated with better nutritional status whereas lower concentrations of high-density lipoprotein (HDL) cholesterol were associated with increased inflammation (Figure 2). 

**Figure 3:**
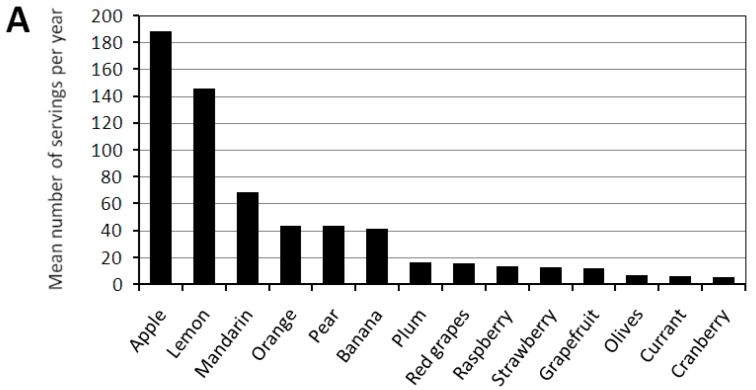

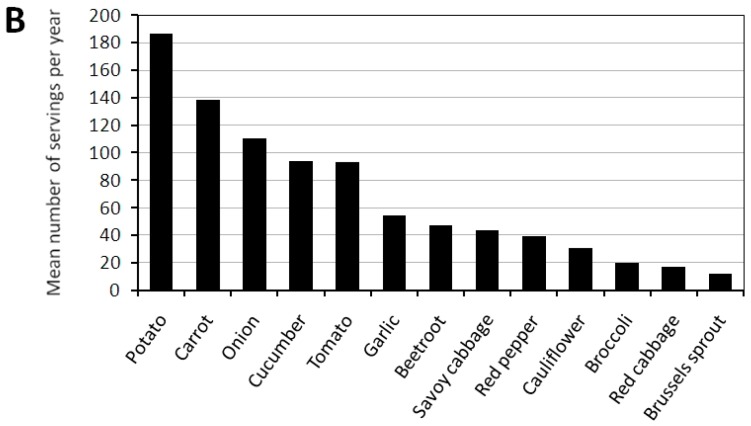
Fruit (**A**) and vegetables (**B**) frequency intake in the hemodialyzed patients’ diet.

Inverse correlation was observed between the intake of exogenous antioxidants in the diet and the concentrations of uric acid (*R* = −0.36; *p* < 0.001) and serum albumin (*R* = −0.22; *p* = 0.036). Neither the selected vegetables nor fruit had a high antioxidant capacity. The most frequently chosen vegetables were: potato, carrot, onion; the most chosen fruits were: apple, lemon, and mandarin (Figure 3). 

## 4. Discussion

Dietary guidelines for hemodialysis patients are considered very restrictive because of the need to limit retained water and to monitor for elevated electrolytes (potassium, sodium, and phosphates). It has been estimated that as many as 50% of hemodialyzed patients develop protein-energy malnutrition [16]. One of useful markers of malnutrition is total cholesterol [17]. However, a large percentage of the study group presented dyslipidemia, and our statistical analysis has not revealed correlations between total cholesterol and malnutrition-inflammation markers.

Systemic inflammation occurs among patients with chronic kidney disease (CKD). Along the drop in glomerular filtration rate (GFR) below 60 mL/min/1.73 m^2^, the severity of inflammation increases with the progression of renal failure [18]. ESRD patients are especially vulnerable to oxidative stress due to the accumulation of uremic toxins and the maintenance hemodialysis treatment itself. Moreover, a wide range of toxic metabolites, such as p-cresol and indoxyl sulphate, can be generated in the fermentation of dietary nutrients in the gut. In CKD, the imbalance of gut microbiota can lead to the translocation of endotoxins to the systemic circulation, which results in immune response, exacerbation of inflammation, and progression of CKD and cardiovascular disease [19,20]. Since uremic toxins and repeated dialyses continue to stimulate the immune system, effective response to circulating pathogens may weaken over time [18]. Cellular immunodeficiency in ESRD can lead to an increased vulnerability to infection progressing either symptomatically or subclinically. For example, one of the frequent problems observed among dialysis patients is periodontal disease [21]. Dental plaque contains bacterial antigens such as lipopolysaccharides or endotoxins capable of inducing a massive local, as well as systemic, inflammation. Other causes of inflammation include bacterial biofilm in dialysis catheters, and catheter-related blood infections, asymptomatic infections of vascular protheses, or various chronic persistent infections such as *Chlamydia pneumoniae*, *Helicobacter pylori*, cytomegalovirus, hepatitis, or tuberculosis [1].

In our study, a higher physical activity of patients was associated with more beneficial values of the laboratory markers of both malnutrition and inflammation, although this association was dependent on the age of patients. This may reflect either the fact that patients with malnutrition and inflammation are less physically active, or the beneficial effect of the moderate physical activity. More studies are needed to assess the causal relationship. Hemodialyzed ESRD patients are generally less physically active than healthy people for several reasons, including anemia, the influence of uremic toxins on muscle cells, secondary parathyroidism, or comorbid cardiovascular disease. Moreover, the dialysis sessions provided three times a week eliminate patients from activity for an average of 12 hours per week. Still, moderate physical activity positively affects malnutrition and inflammation markers, and is an important element of cardiovascular disease prevention. The significance of moderate physical activity should be more emphasized in this group of patients.

ESRD patients are especially vulnerable to elevated oxidative stress, while concomitantly their antioxidant defense mechanisms decline because of vitamin loss during dialysis and dietary restrictions imposed as a preventative measure against hyperkalemia. Increased serum potassium can result in muscle contractions, weakness, paresthesia, and most importantly arrhythmia, heart failure, and eventually death. Due to the life-threatening consequences of hyperkalemia, a low-potassium diet (up to 2–3 g of potassium daily) has been advised. Since potassium is a macronutrient found primarily in plant-based foods, patients significantly reduce fruit and vegetables consumption. Meanwhile, according to Krause’s Food and Nutrition Care Process, it is advised to consume six portions of fruit, vegetables, or juices daily [22]. An increased intake of dietary fiber not only correlates with decreased inflammation (results similar to the group without CKD), but also with decreased mortality [23]. Several processes may lie at the heart of this correlation, i.e., the decrease of the glycemic index of carbohydrates and the concomitant decrease of inflammation; a higher concentration of adiponectin, which is known for its anti-inflammatory properties; the decreased absorption of phenols, indoles, and amines in the large intestine and their increased excretion; a change to the metabolism of intestinal bacteria, and a reduced toxic load [24]. St-Jules et al. [25] indicated that the practice of eliminating plant-derived foods from patients’ diets can be harmful, and should be reevaluated. The consumption of fruit, vegetables, and legumes is closely associated with fiber intake, which has a beneficial effect on gut microbiota, improves peristaltic, and the basic pH of plant foods diminishes metabolic acidosis and can reduce the production of proinflammatory cytokines [23].

It has been insufficiently emphasized that many non-dietary factors influence serum potassium levels. These include prolonged fasting, metabolic acidosis, tissue break-up, constipation or implemented treatment. In the physiological state, 90% of potassium consumed within the diet is excreted by kidneys, and only 10% is excreted via the gastrointestinal tract. However, when the excretory function of the kidneys is impaired, the large intestinal endothelium has the capacity to take over this function to some extent, and excrete potassium: up to 30–40% of potassium consumed with foods can be eliminated via the digestive tract. The consistent prevention of moderate hyperkalemia in ESRD can curtail this compensation mechanism, and pose a risk of postprandial hyperkalemia [26]. In the study conducted in 1960, the defecation of potassium was three times higher in hemodialysis patients (up to 3000 mg/day) than in the healthy population [25]. In the prevention of hyperkalemia, the potassium load can be significantly reduced by means of adequate processing techniques of vegetables, such as double boiling and rinsing without salt. However, the stimulation of intestinal transit and regular bowel movements may be equally important. 

Acid-base homeostasis is another mechanism regulating serum potassium levels. Elevated K^+^ ions are buffered by the exchange of H+ ions between the intracellular and intercellular fluid. The intake of carbohydrates also significantly affects serum potassium concentration. Insulin release stimulates the sodium–potassium pump that facilitates the transport of potassium into the cell [24]. In the ATTICA study, it has been shown that the Mediterranean diet administered in the pre-dialysis period contributes to the increase in the general caloric load of the diet, decreases dyslipidemia, and protects against lipid peroxidation and inflammation, allowing the patients to initiate the dialysis in a satisfactory nutritional status and cardiovascular state [27]. The advantage of the Mediterranean diet stems from its antioxidant and anti-inflammatory properties. It is based on the increased intake of long chain fatty acids from fish and plant oils, legumes, fruits and vegetables, whole food grains, and moderate alcohol consumption. Other nutrients that are equally important for decreasing inflammation are vitamin D, magnesium, and zinc [28,29,30]. Vitamin D modulates the immune system through the VDR receptor in the cell nucleus; however, its activation is impaired when kidney function declines [28]. Magnesium, in turn, takes part in numerous enzymatic processes, suppresses inflammation, and is inversely associated with CRP levels [29]. However, it is important to note that magnesium accumulates in the body in stage 5 CKD patients. Zinc is a cofactor for many enzymes, and its deficiency may influence the production of T lymphocytes taking part in the immune response [30]. The antioxidant enzyme system is another important element in the defense against free radicals; however, it requires the presence of specific microelements that can become deficient in a restrictive, low-calorie diet. The deficiency of elements such as selenium, copper, zinc, and manganese can cause the improper function of endogenous enzymes such as intracellular glutathione peroxidase. What seems to be optimal in the hemodialysis population is a dietary approach that combines high-calorie and protein intake, and the antioxidant properties of fruit and vegetables with a supply of essential microelements, but with acknowledgement of controlled potassium intake. The Mediterranean diet with modifications made specifically for dialysis patients could accommodate this approach.

### Limitations of the Study and Future Research

The authors have made an attempt at creating a questionnaire encompassing lifestyle factors as well as selective dietary exposure in the group of hemodialysis patients. Physical activity has a major influence upon the efficacy of hemodialysis patients’ treatment, and has been placed at the very bottom of the food pyramid as the foundation for a successful dietary intervention. Similarly, other lifestyle factors have an impact upon the patients’ quality of life and implemented diet success rate. However, in future research, it would be appropriate to draw upon the standardized food frequency questionnaires to evaluate fruit and vegetables intake. Moreover, a larger sample size would be warranted, as it would enable a more robust statistical analysis and permit more plausible correlations between the food intake and biochemical parameters.

## 5. Conclusions

There has been an ongoing scientific debate regarding the liberalization of dietary guidelines issued to patients treated with maintenance hemodialysis, which thus far have primarily concentrated on the decrease of potassium, phosphorus, and water intake. However, what has been recently proposed instead is an increase in dietary fiber, antioxidants, and phytochemicals, all of which have a big impact on the improvement of the nutritional status and cardiovascular state of a patient [31]. The reactions of low-molecular exogenous antioxidants, such as vitamin C, E, or carotenoids, are less specific than endogenous antioxidant enzymes, but at the same time, they comprise a more universal defense system against the reactive oxygen species. The best source of vitamins and polyphenols are fruit and vegetables that are supplied on a day-to-day basis; therefore, close cooperation between hemodialysis patients and a dietician is essential in securing a tailored dietary intervention. The approach should target an individual’s nutritional status, his/her residual renal function, and any MIA components and other comorbidities while taking into account any additional laboratory test results. The widely recognized Kidney Disease Outcomes Quality Initiative (KDOQI) guideline number 8 recommends that every patient and/or caretaker receive intensive nutrition counseling at the start of dialysis [11]. Frequent follow-up would allow for dietary adjustments to maximize nutrition and help prevent malnutrition-inflammation-atherosclerosis syndrome. 

## Figures and Tables

**Table 1:** Demographic characteristics, lifestyle, and overall fruit and vegetables consumption frequency of the study group.

Characteristic	Whole Study Group (*n* = 98)	Men (*n* = 60)	Women (*n* = 38)	*p*-Value
Age, years	62 ± 14	61 ± 16	65 ± 11	0.2
Duration of dialysis, months	63 (35–144)	60 (36–108)	84 (36–192)	0.3
Residence				
Country, *n*/%	34/35	19/32	15/39	0.9
Town <100,000 inhabitants, *n*/%	10/10	6/10	4/11	
City >100,000 inhabitants, *n*/%	54/55	35/58	19/50	
Body weight, kg	71.6 ± 16.3	76.0 ± 14.4	64.8 ± 16.8	<0.001
BMI, kg/m^2^	25.2 ± 5.0	25.7 ± 4.4	24.5 ± 5.8	0.3
Active smoking, *n*/%	17/17	12/20	5/8	0.3
Sleep				
≤6 h/24 h, *n*/%	32/33	17/28	15/39	0.6
7–8 h/24 h, *n*/%	48/49	30/50	18/47	
9 h/24 h, *n*/%	15/15	10/17	5/13	
Physical activity				
Low, *n*/%	51/52	28/47	23/61	0.3
Moderate, *n*/%	44/45	29/48	15/39	
Dietary supplements’ use, *n*/%	31/32	18/30	13/34	0.7
Vegetables frequency				
>2 portions/day, *n*/%	3/3	2/3	1/3	0.8
1–2 portions/day, *n*/%	47/48	30/50	17/45	
A few portions/week, *n*/%	36/37	20/33	16/42	
<1 portion/week, *n*/%	6/6	3/5	3/8	
Fruit frequency				
>2 portions/day, *n*/%	5/5	0	5/13	0.005
1–2 portions/day, *n*/%	52/53	34/57	18/47	
A few portions/week, *n*/%	20/20	9/15	11/29	
<1 portion/week, *n*/%	16/16	13/22	3/8	
Fruit and vegetables processing				
Eating raw, *n*/%	36/37	24/40	12/32	0.6
Cooking, *n*/%	42/43	24/40	18/47	
Cooking with water change, *n*/%	15/15	8/13	7/18	

**Table 2:** The characteristic of the selected markers of malnutrition and inflammation.

Study Parameters (Degree of Malnutrition)	Whole Study Group (*n* = 98)	Men (*n* = 60)	Women (*n* = 38)	*p*-Value
Albumin, g/L	39.4 (36.4–42.8)	40.7 (37.4–43.1)	37.8 (36.1–40.9)	0.022
30–34 g/L (mild), *n*/%	10/10	6/10	4/11	0.5
21–29 g/L (moderate), *n*/%	4/4	2/3	2/5	
<21 g/L (severe), *n*/%	0	0	0	
Prealbumin, g/L	0.27 (0.22–0.32)	0.28 (0.24–0.33)	0.25 (0.20–0.30)	0.063
0.10–0.17 g/L (mild), *n*/%	8/8	4/7	2/5	0.4
0.05–0.09 g/L (moderate), *n*/%	0	0	2/5	
<0.05 g/L (severe), *n*/%	0	0	0	
GPS 0, *n*/%	56/57	36/60	20/53	0.5
GPS 1, *n*/%	25/25	16/27	9/24	
GPS 2, *n*/%	11/11	5/8	6/16	
CRP/PRE	0.019 (0.008–0.059)	0.016 (0.006–0.052)	0.024 (0.011–0.072)	0.09
CRP, mg/L	5.7 (2.2–13.7)	5.7 (1.7–12.1)	5.9 (2.9–16.7)	0.3
CRP >5 mg/L, *n*/%	52/53	30/50	22/58	0.3

GPS—Glasgow Prognostic Score; PRE—prealbumin; CRP—C-reactive protein.

**Table 3:** The correlations between the selected markers of malnutrition and inflammation, as well as total cholesterol, and hemoglobin (HGB).

	GPS	CRP/PRE	CRP	Prealbumin	Total Cholesterol	HGB
Albumin	−0.52 *	−0.46 *	−0.42 *	0.59 *	−0.04 ^NS^	0.36 *
Prealbumin	−0.61 *	−0.64 *	−0.50 *	-	0.24 ^NS^	0.17 ^NS^
CRP	0.80 *	0.97 *	-	-	0.14 ^NS^	−0.11 ^NS^
CRP/PRE	0.80 *	-	-	-	0.11 ^NS^	−0.14 ^NS^
GPS	-	-	-	-	0.06 ^NS^	−0.10 ^NS^

^NS^—Non-significant; *—*p* < 0.001; HGB—hemoglobin.

**Table 4:** The results of simple and age-adjusted logistic regression performed to identify predictors of malnutrition and inflammation.

	Albumin ≤ 34 g/L	Prealbumin ≤ 0.17 g/L	CRP > 5 mg/L	CRP/PRE > 0.019	GPS > 0
	Simple odds ratio (95% confidence interval)				
Age, per 1 year	1.03 (0.99–1.08) ^NS^	1.01 (0.96–1.06) ^NS^	1.03 (0.99–1.06) ^NS^	1.04 (1.003–1.07) *	1.05 (1.01–1.09) **
Female sex	1.27 (0.39–4.08) ^NS^	1.74 (0.40–7.60) ^NS^	1.52 (0.64–3.64) ^NS^	1.92 (0.81–4.57) ^NS^	1.29 (0.54–3.07) ^NS^
Physical activity	0.32 (0.08–1.32) ^NS^	0.63 (0.14–2.87) ^NS^	0.52 (0.22–1.22) ^NS^	0.34 (0.14–0.82) *	0.37 (0.15–0.92) *
Fruit frequency ≥1 portion/day	0.26 (0.07–0.96) *	0.62 (0.14–2.72) ^NS^	1.57 (0.65–3.79) ^NS^	1.00 (0.42–2.39) ^NS^	1.19 (0.49–2.90) ^NS^
Vegetables’ frequency ≥1 portion/day	0.51 (0.15–1.81) ^NS^	0.80 (0.18–3.48) ^NS^	1.50 (0.62–3.60) ^NS^	1.09 (0.46–2.58) ^NS^	0.82 (0.34–1.99) ^NS^
	Age-adjusted odds ratio (95% confidence interval); *p*-value				
Physical activity	-	-	-	0.40 (0.16–1.01) ^NS^	0.46 (0.18–1.20) ^NS^

^NS^—Non-significant; *—*p* < 0.05; **—*p* < 0.01.

**Table 5:** The results of laboratory tests in the study group.

Laboratory Test	Results	Reference Range	Results < Reference Range, *n*/%	Results > Reference Range, *n*/%
Complete blood counts				
WBC, ×10^3^/µL	6.5 (5.1–7.8)	4.0–10.0	8/8	8/8
RBC, ×10^6^/µL				
Men	3.8 ± 0.6	4.5–6.5	55/92	0
Women	3.6 ± 0.5	3.5–5.0	19/50	0
HGB, g/dL				
Men	11.3 ± 1.5	12.0–17.0	43/72	0
Women	10.7 ± 1.2	11.0–15.0	21/55	0
HCT, %				
Men	35.0 ± 4.6	40.0–54.0	53/88	0
Women	33.1 ± 3.5	37.0–47.0	31/82	0
MCV, fL	92.9 ± 6.0	82.0–92.0	3/3	53/54
MCH, pg	30.0 ± 2.0	27.0–31.0	8/8	29/30
MCHC, g/dL	32.3 ± 1.1	32.0–36.0	31/32	0
PLT, × 10^3^/µL	203.8 ± 67.4	125.0–340.0	11/11	3/3
RDW-CV, %	15.2 (14.0–16.2)	11.0–15	0	52/53
Biochemistry				
Sodium, mmol/L	137 (136–139)	136–145	24/24	0
Potassium, mmol/L	5.1 ± 0.9	3.5–5.1	1/1	48/49
Calcium, mmol/L	1.89 (1.18–2.27)	2.15–2.55	63/64	5/5
Phosphate, mmol/L	2.50 (1.65–4.50)	0.81–1.45	3/3	80/82
Iron, µmol/L	11.80 (9.12–15.0)	5.83–34.50	3/5	0
UIBC, µmol/L				
Men	30.7 ± 10.8	22.3–61.7	10/25	0
Women	30.8 ± 10.7	24.2–70.1	7/26	0
TIBC, µmol/L	42.2 ± 10.3	40.8–76.6	38/53	0
Urea, mmol/L	20.85 ± 7.90	2.76–8.07	1/1	88/90
Total cholesterol, mmol/L	4.2 ± 1.4	3.2–5.2	11/25	11/25
HDL-C, mmol/L	1.0 ± 0.3	0.9–3.0	13/30	0
LDL-C, mmol/L	2.3 ± 1.1	0.2–3.4	0	8/18
Triglycerides, mmol/L	1.5 (1.2–2.2)	0.2–2.3	0	9/20

WBC—white blood cells; RBC—red blood cells; HGB—hemoglobin; HCT—hematocrit; MCV—mean cell volume; MCH—mean corpuscular hemoglobin; MCHC—mean corpuscular hemoglobin concentration; PLT—platelets; RDW-CV—red cell distribution width; UIBC—latent iron binding capacity; TIBC—total iron binding capacity; LDL-C—low density lipoprotein cholesterol; HDL-C—high density lipoprotein cholesterol.
